# Design of Aromatic Interaction Networks in a Protein Cage Modulated by Fluorescent Ligand Binding

**DOI:** 10.1002/advs.202417030

**Published:** 2025-02-20

**Authors:** Yuki Hishikawa, Taiga Suzuki, Basudev Maity, Hiroki Noya, Michito Yoshizawa, Asuka Asanuma, Yuri Katagiri, Satoshi Abe, Satoru Nagatoishi, Kouhei Tsumoto, Takafumi Ueno

**Affiliations:** ^1^ School of Life Science and Technology Institute of Science Tokyo Nagatsuta‐cho 4259, Midori‐ku Yokohama 226‐8501 Japan; ^2^ Laboratory for Chemistry and Life Science Institute of Integrated Research Institute of Science Tokyo Nagatsuta‐cho 4259, Midori‐ku Yokohama 226‐8501 Japan; ^3^ The Institute of Medical Science The University of Tokyo Shirokanedai 4‐6‐1, Minato‐ku Tokyo 108‐8639 Japan; ^4^ Department of Bioengineering School of Engineering The University of Tokyo Hongo 7‐3‐1, Bunkyo‐ku Tokyo 113‐8656 Japan; ^5^ Research Center for Autonomous Systems Materialogy (ASMat) Institute of Integrated Research Institute of Science Tokyo Nagatsuta‐cho 4259, Midori‐ku Yokohama 226‐8501 Japan

**Keywords:** aromatic interaction, fluorescence, ligand binding, protein cage, protein design

## Abstract

Dynamic behavior of proteins, such as orientation changes of aromatic residues, plays an important role in controlling biomolecular functions. Protein design that can precisely control such dynamic behavior at the atomic level is a challenging issue. The study reports the development of a system capable of orientational changes of aromatic side chains upon ligand binding. Aromatic pockets are constructed on the inner surfaces of protein cages to bind polycyclic aromatic fluorescent molecules to the targeted position by π–π stacking interactions. X‐ray crystal structural analysis indicated the cooperative orientation changes of the aromatic clusters around the pocket triggered by the ligand binding. A comparison of various ligands shows that the movement of aromatic clusters can be controlled depending on the ligand structures. Fluorescence quantum yield and fluorescence lifetime are enhanced due to isolation of the fluorescent molecules in an aromatic pocket. These findings provide an understanding of the unique molecular behavior and fluorescence properties of ligands due to the assembly of aromatic residues and a guideline for developing dynamically controlled supramolecular biomaterials.

## Graphical abstract

The study presents a novel system designed to control protein dynamics by orientational changes of aromatic residues triggered by ligand binding. By creating aromatic pockets within protein cages and binding fluorescent molecules via π–π interactions, the study achieves cooperative conformational change verified through X‐ray crystallography. The fluorescence properties of the ligands are enhanced, offering insights into dynamic biomaterial design.

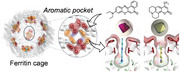

## 1 Introduction

Aromatic interactions are fundamental to the molecular architecture and functionality of biological systems, facilitating molecular recognition,^[^ ^1^ ^]^ protein folding,^[^ ^2^ ^]^ stabilization of protein complexes^[^ ^3^ ^]^ and nucleic acids. Multiple aromatic residues often form clusters at ligand‐recognition sites of proteins.^[^ ^4^ ^]^ The dynamic behavior of aromatic residues, such as side chain reorientation, is potentially linked to RNA regulatory mechanisms,^[^ ^5^ ^]^ enzymatic catalysis,^[^ ^6^ ^]^ membrane protein–ligand interactions,^[^ ^7^ ^]^ and allostery of hemoglobin.^[^ ^8^ ^]^ These instances underscore the significance of the dynamic interplay between aromatic interactions and conformational dynamics in molecular binding. Advances in NMR^[^ ^9^ ^]^ and X‐ray crystallography^[^ ^10^ ^]^ have begun to unravel the rotational behaviors of aromatic residues, spotlighting the utility of aromatic interactions in protein design.^[^ ^11^ ^]^ Although the protein engineering techniques have successfully constructed aromatic clusters with enhanced thermostability by introducing several aromatic residues in peptides, alpha‐helix bundles, and protein cages,^[^ ^11^, ^12^ ^]^ a design for controlling the protein dynamics at the sidechain level remains challenging.

**Figure 1:**
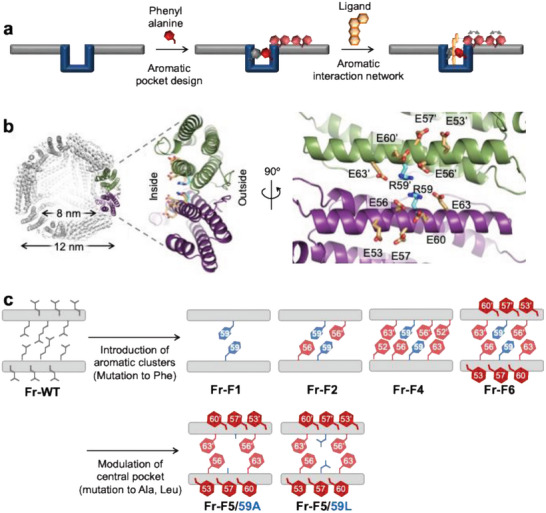
Design of a molecular system for conformational changes of aromatic sidechains triggered by fluorophore‐binding in protein cages. a) Concept of constructing aromatic pocket at the two‐fold symmetric interface. Binding of the key molecule to the pocket triggers an orientation change of a Phe sidechain, which induces the conformational changes of the neighboring Phe sidechains. Orientation changes of the sidechains are not induced by small molecules that differ in shape from the key molecule. b) The structure of ferritin cage and the two‐fold symmetric interface (PDB ID: 1DAT). c) Schematic diagram for design of ferritin mutants by amino acid substitution at the two‐fold symmetric interface.

To address this challenge, we design ligand‐binding pockets surrounded by engineered aromatic clusters within a protein cage. This setup is expected to cooperatively alter and visualize the movements of aromatic side chains upon ligand binding (**Figure** **1**a). As a scaffold for aromatic cluster design, we chose ferritin as a protein cage platform with a 12 nm outer diameter and an 8 nm inner cavity, composed of 24 subunits. Ferritin's structural robustness and versatility have facilitated the encapsulation of diverse entities, including metal ions, nanoparticles, organometallic complexes, nucleic acids, and pharmaceutical molecules.^[^ ^13^ ^]^ While interactions at the two‐fold symmetric interfaces of ferritin have been implicated in the binding of specific molecules (ferrocene derivatives, halothane, and isoflurane^[^ ^14^ ^]^), the precise engineering of molecular binding pockets in the cage and the detailed elucidation of amino acid conformational dynamics have remained elusive.

Herein, we present the construction of an aromatic cluster with an artificial molecular binding pocket at the two‐fold symmetric interface of ferritin and explore its ligand‐binding characteristics. We developed a system that uses ligand binding as a driving force to induce and propagate changes in the orientation of multiple aromatic side chains. To have more insight on Phe side chain orientation, we designed variants with Phe substitutions ranging from one to six residues. X‐ray crystallography confirmed the binding of aromatic fluorescent molecules to the pocket and the cooperative reorientation of multiple Phe side chains. We also examined the relationship between side chain orientation changes and substrate binding by substituting pocket‐forming amino acids with aromatic (Phe) or hydrophobic (Ala, Leu) residues, observing shape‐dependent conformational changes in surrounding Phe residues. This study enhances our understanding of the unique molecular behaviors elicited by aromatic clusters and offers guidelines for the dynamic control of protein side chains and developing supramolecular machinery.

## 2 Results

### 2.1 Design of Aromatic Clusters with Ligand‐Binding Pockets

We hypothesized that by artificially designing ligand binding sites and arranging aromatic residues around them, ligand binding could be the driving force to dynamically control the orientation of the aromatic side chains (Figure 1a). To prove this concept through protein engineering, we focused on a two‐fold symmetric inter‐subunit interface of a recombinant horse spleen apo‐L ferritin (Fr) as a target region for mutagenesis (Figure 1b). Two residues of Arg59 are located at the center of this interface. Their side chains are aligned nearly parallel to each other (Figure 1b). We previously demonstrated that the substitution of Arg59 with Phe59 leads two phenyl rings to be aligned parallel with a central distance of 7.8 Å, forming an aromatic pocket between the subunits (PDB ID: 8H8L, ref. [12]) We expect that this pocket can immobilize aromatic molecules via π–π stacking interactions. To create the ligand‐binding pocket, we first designed a mutant **Fr‐F1** (Fr‐R59F) in which Arg59 was replaced by Phe59 (Figure 1c). Next, we replaced Glu residues located near residue 59 with Phe residues to form an aromatic cluster surrounding the Phe59 pocket. The resulting mutants were named **Fr‐F2** (Fr‐E56F/**R59F**), **Fr‐F4** (Fr‐R52F/E56F/**R59F**/E63F), and **Fr‐F6** (Fr‐E53F/E56F/E57F/R59F/E60F/E63F), where *i* in **Fr‐F*i*** represents the number of newly introduced Phe residues per ferritin monomer (Figure 1c, upper). To modulate the shape of the central pocket, we prepared **Fr‐F5/59A** (Fr‐E53F/E56F/E57F/**R59A**/E60F/E63F) and **Fr‐F5/59L** (Fr‐E53F/E56F/E57F/**R59L**/E60F/E63F) in which residue 59 was replaced with Ala or Leu (Figure 1c, lower).

The designed mutants were expressed in *E. coli* and purified according to the reported procedures.^[^ ^15^ ^]^ The mass of mutant monomers were confirmed by matrix‐assisted laser desorption ionization‐time of flight mass spectrometry (MALDI‐TOF‐MS) (Figure S1a, Supporting Information). The 24‐mer cages of the mutants were confirmed by native‐PAGE, and dynamic light scattering (DLS) (Figure S1b,c, Supporting Information). The thermostability of the mutants was evaluated by differential scanning calorimetry (DSC). The DSC data shows that all mutants have melting temperatures exceeding 120 °C, indicating the high structural stability of the mutants (*T*_m_ = 125.9, 125.1, 126.9, 122.2, 123.8, and 123.2 °C for **Fr‐F1**, **Fr‐F2**, **Fr‐F4**, **Fr‐F6**, **Fr‐F5/59A**, and **Fr‐F5/59L**; Figure S2, Supporting Information).

**Figure 2:**
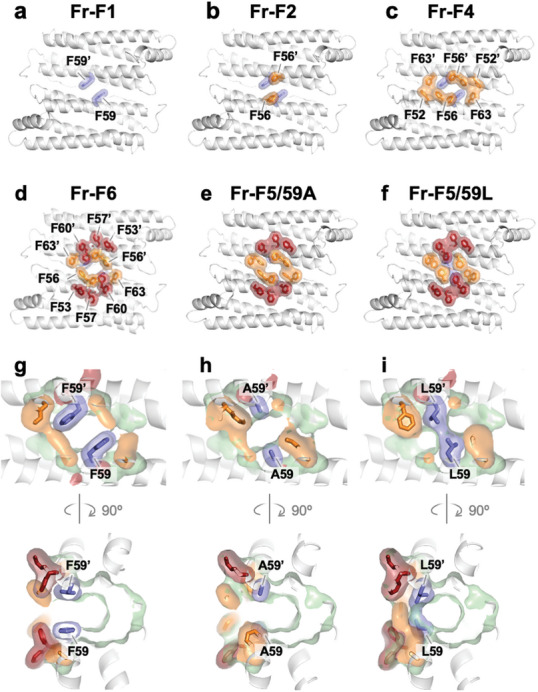
X‐ray crystal structures of apo‐ferritin mutants. a–f) Structures of the dimers. (a) **Fr‐F1**. (b) **Fr‐F2**. (c) **Fr‐F4**. (d) **Fr‐F6**. (e) **Fr‐F5/59A**. (f) **Fr‐F5/59L**. g–i) Structures of the pockets located at the residue number 59. g) **Fr‐F6**. h) **Fr‐F5/59A**. i) **Fr‐F5/59L**. In panel (i), the entrance to the pocket is blocked by side chains of the inter‐subunit Leu59 residues.

The X‐ray crystal structures of the mutants were determined at 1.50–1.53 Å resolution (Table S1, Supporting Information for crystallographic statistics, Supporting Information). The analysis shows the formation of a pocket at residue 59 for **Fr‐F1**, **Fr‐F2**, **Fr‐F4**, and **Fr‐F6** (**Figure** **2**a–d). Two phenyl rings of Phe59–Phe59’ are parallel at the subunit interface, giving a ring centroid distance of 7.8 Å (Figure 2g). Within the vicinity of pocket F59, an intricate network of aromatic interactions is established, comprising three phenylalanine (Phe) residues (F52, F56, and F63) in **Fr‐F4** and five Phe residues (F53, F56, F57, F60, and F63) in **Fr‐F6** (Figure 2c,d). In the case of **Fr‐F5/59A**, the replacement of Phe59 with alanine (Ala) results in an expanded pocket size (Figure 2e,h). Conversely, in **Fr‐F5/59L**, the introduction of leucine (Leu) imposes steric hindrance, effectively obstructing the pocket's entrance (Figure 2f,i). These variances in pocket morphology among the variants are anticipated to impact ligand binding affinity and specificity. Consequently, the array of mutants possessing the F59–F59' pocket (**Fr‐F1**, **Fr‐F2**, **Fr‐F4**, **Fr‐F6**) could be utilized for the binding of aromatic molecules, while **Fr‐F5/59A** and **Fr‐F5/59L** may serve as comparative controls within this experimental framework.

### 2.2 Incorporation of Fluorescent Ligands into Protein Cages

**Figure 3:**
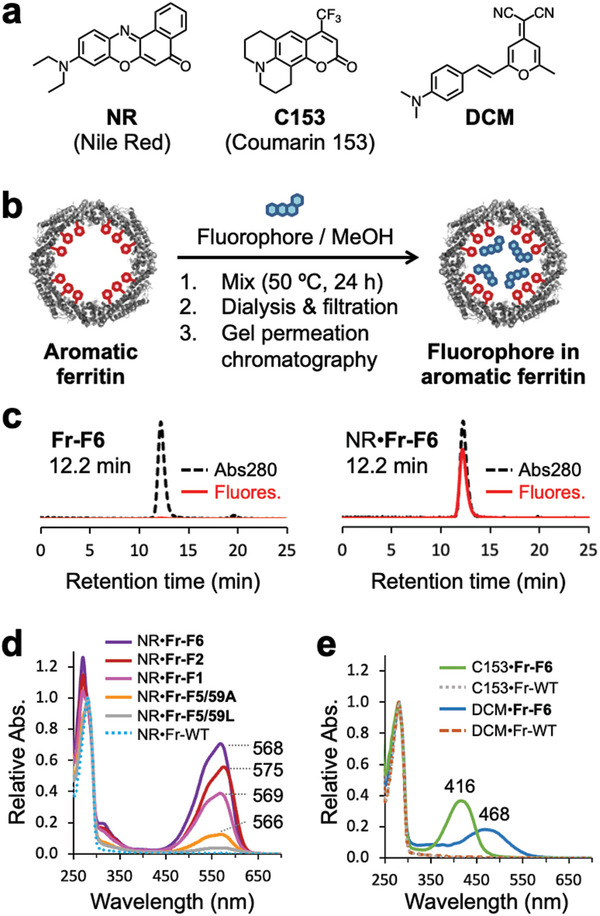
Incorporation of fluorophores into ferritin cages. a) Structures of fluorophores: Nile red (NR), Coumarin 153 (C153), and 4‐(dicyanomethylene)‐2‐methyl‐6‐(4‐dimethylaminostyryl)‐4H‐pyran (DCM). b) Reaction scheme for incorporation. c) HPLC elution profile before and after reaction with fluorophores. The elution was monitored by UV absorbance at 280 nm (dashed black line) and fluorescence at *λ*_ex_ = 535 nm and *λ*_em_ = 600 nm (red solid line). d) UV–vis absorption spectra of **NR•Fr‐F6**, **NR•Fr‐F1**, **NR•Fr‐F5/59A**, **NR•Fr‐F5/59L**, and NR•Fr‐WT. (e) **C153•Fr‐F6**, C153•Fr‐WT, **DCM•Fr‐F6**, and DCM•Fr‐WT.

For binding of ligands to the constructed pocket, we selected polycyclic aromatic fluorescent molecules, Nile red (NR), Coumarin 153 (C153), and 4‐(dicyanomethylene)‐2‐methyl‐6‐(4‐dimethylaminostyryl)‐4H‐pyran (DCM) for four reasons (**Figure** **3**a). First, these molecules have been previously encapsulated into synthetic aromatic micelles in aqueous media.^[^ ^16^, ^17^ ^]^ Second, these molecules have multiple aromatic rings in their structures, which would promote incorporation into the protein cages through π–π interactions with aromatic residues. Third, the encapsulation can be assessed owing to their absorbance in the visible region and low solubility in water. Fourth, fluorescence measurement can be used to explore the characteristics of designed pockets since the fluorescent properties of ligands reflect the surrounding environment. The fluorophores in methanol solution (NR, C153, and DCM, 100 equivalents against protein cage) were mixed with an equal volume of ferritin buffer solution (50 mM Tris‐HCl, pH 8, 150 m NaCl) and incubated at 50 °C for 24 hours (Figure 3b). The reaction mixture was dialyzed against tris buffer to remove free fluorophores that were not encapsulated in the cage. The elution profiles of gel permeation chromatography indicate the successful incorporation of the fluorophores into the protein cages (Figure 3c). The absorption spectra also confirm the incorporation by showing bands corresponding to the fluorophores (566–575 nm for Nile red, 416 nm for C153, and 468 nm for DCM), as well as bands at 280 nm corresponding to the proteins (Figure 3d,e). In contrast, Fr‐WT shows no absorption bands corresponding to the fluorescent molecules (Figure 3d). These results indicate that the engineered ferritin variants enable the encapsulation of fluorescent molecules.

The number of fluorophores inside the ferritin cage was quantified by UV–vis absorption spectroscopy and Lowry assay (Figure 3d,e, and **Table** **1**). The largest number was obtained for **NR•Fr‐F6**, showing 10.4 molecules of NR are encapsulated per protein cage. This amount is in good agreement with the number of the pockets (12) at the two‐fold symmetric interface in the ferritin cage, suggesting that 12 pockets of **Fr‐F6** are occupied by approximately one molecule of NR. The amount of loaded NR increased as the number of introduced Phe residues increased, as shown by comparing **Fr‐F6** and **Fr‐F1**. Replacing Phe59 with Ala or Leu significantly reduced the incorporation amount of NR (1.4 molecules for **Fr‐F5/59A** and 0.4 molecules for **Fr‐F5/59L**). These results imply that the NR–F59 binding is promoted by the surrounding Phe residue clusters. The loaded amount of C153 and DCM into **Fr‐F6** was 7.2 and 0.9 molecules per cage, respectively. The results suggest that the molecular structure of NR fits into the pocket of **Fr‐F6**, compared to C153 and DCM.

**Table 1:** Number of fluorescent molecules incorporated into a ferritin cage.

Sample	Fluorophore/Fr‐cage^a^ ^)^
NR•Fr‐F1	4.7 ± 1.6
NR•Fr‐F2	6.4 ± 1.5
NR•Fr‐F4	6.2 ± 1.4
NR•Fr‐F6	10.4 ± 3.1
NR•Fr‐F5/59A	1.3 ± 0.2
NR•Fr‐F5/59L	0.3 ± 0.1
NR•Fr‐WT	Not detected^b^ ^)^
C153•Fr‐F6	7.2 ± 0.9
C153•Fr‐WT	Not detected^b^ ^)^
DCM•Fr‐F6	0.9 ± 0.1
DCM•Fr‐WT	Not detected^b^ ^)^

^a)^ The number of fluorophores incorporated into the ferritin cages was determined from the UV–vis absorbance and Lowry assay. Data are presented as mean ± standard deviation (*n* = 3).

^b)^ Absorption corresponding to fluorophores was not detected from the UV–vis absorption spectrum after reaction of Fr‐WT with the fluorophores.

### 2.3 X‐Ray Crystal Structures of Ferritin in Complex with Fluorescent Ligands

**Figure 4:**
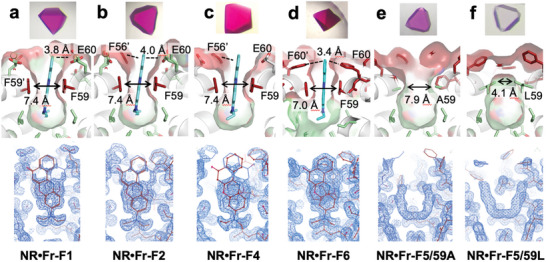
X‐ray crystal structures of ferritin‐NR complexes. a–f) Crystal images of NR complexes (upper), NR binding modes in the molecular pockets at the twofold symmetric interfaces (middle), and 2Fo‐Fc maps (1σ) (lower). a) **NR•Fr‐F1**. b) **NR•Fr‐F2**. c) **NR•Fr‐F4**. d) **NR•Fr‐F6**. e) **NR•Fr‐F5/59A**. f) **NR•Fr‐F5/59L**. Since NR is located at the twofold symmetric interface, for clarity, only one molecule is shown in the middle panel on each picture.

**Figure 5:**
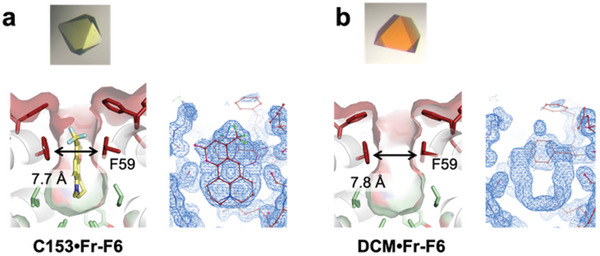
X‐ray crystal structures of ferritin‐C153 and DCM complexes. a,b) Crystal images of C153 and DCM complexes (upper), ligand binding modes in the molecular pockets at the twofold symmetric interfaces (lower left), and 2Fo‐Fc maps (1σ) (lower right). (a) **C153•Fr‐F6**. (b) **DCM•Fr‐F6**.

To examine the binding modes of fluorescent molecules and the structural changes of surrounding amino acids, we determined the X‐ray crystal structures of ferritin‐fluorescent molecule complexes at resolutions of 1.50–1.66 Å (**Figures** **4** and **5**; Figure S3, Tables S2 and S3, Supporting Information for crystallographic statistics). Analysis of the 2Fo‐Fc maps revealed electron densities corresponding to Nile Red (NR) at the two‐fold symmetric interfaces of **NR•Fr‐F1**, **NR•Fr‐F2**, **NR•Fr‐F4**, and **NR•Fr‐F6**, observed as overlapping densities due to the interface symmetry (**Figure** **4**a–d; Figure S3, Supporting Information). NR was positioned between two F59 residues, forming π–π stacking interactions in an F59–NR–F59' configuration (Figure 4a–d). Anion‐π interactions between NR and E60 in **NR•Fr‐F1** and **NR•Fr‐F2**, and CH‐π interactions between NR and F60 in **NR•Fr‐F6**, were identified, indicating NR's stabilization in the F59–F59' pocket via multiple non‐covalent interactions.

For **NR•Fr‐F5/59A** and **NR•Fr‐F5/59L**, no assignable electron density of NR were observed in the 2*F*_o_‐*F*_c_ maps (Figure 4e,f; Figure S3, Supporting Information). Difference density (*F*_o, NR –_ *F*_o,apo_) features between apo form and NR loaded form showed very weak density near F59 in **NR•Fr‐F5/59A** suggesting poor NR binding at this site (Figure S3b, Supporting Information, green mesh). This is also reflected in the color of the crystal which showed very light purple color unlike **NR•Fr‐F6**. Although there was sufficient space for NR in the A59 pocket (distance between A59–A59' 7.9 Å), the substitution of F59 with A59 excluded π–π stacking interactions at the site. As a result, NR has poor affinity to bind at this site in **Fr‐F5/59A** (Figure S3b, Supporting Information). In contrast, no electron density attributable to NR was observed in the difference Fourier map analysis of **NR•Fr‐F5/59L** (Figure S3c, Supporting Information), likely due to the entrance being blocked by L59 (distance between L59–L59' 4.1 Å), preventing NR entry due to steric hindrance. These crystallographic observations of NR electron densities and binding modes are generally consistent with the quantification of NR encapsulation (Table 1) and the trends observed in UV‐vis spectra (Figure 3d).

Upon comparing the protein structures before and after Nile Red (NR) encapsulation, orientational changes of Phe side chains were observed in **NR•Fr‐F6** due to NR binding (Figure S3a, Supporting Information). In **NR•Fr‐F6**, compared to **Fr‐F6** without NR, the occupancy of the alternate conformation of F60 and F56 changed from 0.3 to 0.7 and 0.3 to 0.5, respectively. The dihedral angles χ1 (defined by the N–Cα–Cβ–Cγ torsion) of the F56 and F60 side chains, which directly interact with NR, changed by 219° and 102°, respectively (Figure S3a, Supporting Information). Since the residue F57 and F53 have tendency to form double conformation having weak density of the alternate confer, this might be an indication that the rotational movements of F56 and F60 upon NR binding propagate to F57 and F53. In **NR•Fr‐F4**, a change in the χ1 angle of F56 was noted among the four introduced Phe residues, but no significant orientation changes were observed for the other Phe residues (F52, F59, F63). In **NR•Fr‐F5/59A**, an orientation change of F63 was detected following NR encapsulation, but no changes were observed for F53, F56, F57, and F60 (Figure S3b, Supporting Information). Similarly, in **NR•Fr‐F5/59L**, no orientation changes were detected for the five introduced Phe residues (Figure S3c, Supporting Information). These variant comparisons demonstrate that tight pocket fixation of NR via sandwich‐type π–π stacking interactions of F59–NR–F59' is crucial for the orientation control of surrounding aromatic residues.

Having observed the induction of Phe side‐chain orientation changes by NR in **Fr‐F6**, we next investigated whether other aromatic molecules (C153, DCM) could be accommodated within the pocket and drive orientation changes. Structural analysis of **C153•Fr‐F6** revealed distinct electron densities between F59–F59', confirming the stabilization of C153 within the two‐fold symmetric interface pocket through F59–C153–F59' π–π stacking interactions (**Figure** **5**a). In contrast, no corresponding electron density for DCM was observed in **DCM•Fr‐F6** (Figure 5b), aligning with quantitative results showing greater incorporation of C153 into **Fr‐F6** compared to DCM (Table 1). The observed density is similar to apo protein structure or other ferritin structures where no ligand was mixed (Figure 5b; Figure S4, Supporting Information). C153's polycyclic aromatic structure likely facilitates its recognition by F59–F59' through planar aromatic interactions. In contrast, DCM's structure, featuring a –C = C– bridge separating the benzene and pyran rings, likely hinders the formation of π–π stacking interactions with F59–F59'. Comparative crystallographic analysis before and after encapsulation of C153 and DCM showed no significant changes in Phe side‐chain orientations in either **C153•Fr‐F6** or **DCM•Fr‐F6** (Figure S3d,e, Supporting Information). These findings suggest that changes in Phe orientation depend on the molecular shape of the ligand as we found NR binding altered the changes in Phe residues.

### 2.4 Fluorescence Characterization

Fluorescence measurements were conducted to evaluate the characteristics of the designed aromatic clusters and pockets, as the fluorescent properties of ligands reflect their local environment. Specifically, fluorescence quantum yields and lifetimes were analyzed (**Table** **2**; Figure S5, Supporting Information). The quantum yield of Nile Red (NR) significantly increased from 0.50 in 100% methanol (MeOH) and 0.38 in a 50% MeOH‐water mixture to 0.88–0.95 upon encapsulation within **Fr‐F1**, **Fr‐F2**, **Fr‐F4**, and **Fr‐F6**. Furthermore, NR's fluorescence lifetime extended from 2.87 ns in organic solvent and 1.26 ns in aqueous‐organic mixture to 5.23–5.75 ns within these ferritin variants (Figure S5a,b, Supporting Information). **NR•Fr‐F5/59A** and **NR•Fr‐F5/59L** exhibited quantum yields of 0.79 and 0.65, respectively, higher than NR in MeOH but less pronounced compared to other variants. This pattern was also observed in their fluorescence lifetimes, suggesting that the immobilization of NR through F59–F59' interaction significantly contributes to enhanced fluorescence characteristics.

**Table 2:** Fluorescence properties of fluorescent molecules in ferritin or organic solvents.

Sample	*Φ*_F_ ^a^ ^)^	<τ> (ns)^b^ ^)^	*χ* ^2b)^
NR•Fr‐F1	0.94 ± 0.06	5.54 ± 0.03	1.00^c^ ^)^
NR•Fr‐F2	0.95 ± 0.05	5.75 ± 0.02	1.04^c^ ^)^
NR•Fr‐F4	0.92 ± 0.03	5.23 ± 0.01	1.03^c^ ^)^
NR•Fr‐F6	0.88 ± 0.06	5.40 ± 0.05	1.17^c^ ^)^
NR•Fr‐F5/59A	0.79 ± 0.05	4.88 ± 0.01	1.01^c^ ^)^
NR•Fr‐F5/59L	0.65 ± 0.07	4.42 ± 0.00	1.10^d^ ^)^
NR (MeOH)	0.50 ± 0.01	2.87 ± 0.02	1.01^c^ ^)^
NR (50% MeOH, Tris)	0.38 ± 0.06	1.26 ± 0.01	1.04^c^ ^)^
C153•Fr‐F6	0.73 ± 0.02	6.89 ± 0.03	1.08^d^ ^)^
C153 (MeOH)	0.48 ± 0.04	4.01 ± 0.02	1.01^c^ ^)^
C153 (50% MeOH, Tris)	0.43 ± 0.02	2.77 ± 0.06	1.11^c^ ^)^
DCM•Fr‐F6	0.41 ± 0.03	2.98 ± 0.04	0.98^d^ ^)^
DCM (MeOH)	0.35 ± 0.01	1.38 ± 0.02	0.97^d^ ^)^
DCM (50% MeOH, Tris)	0.18 ± 0.02	0.60 ± 0.02	1.03^d^ ^)^

^a)^ Fluorescence quantum yield. *λ*_ex_ = 535 nm for Nile red, *λ*_ex_ = 394 nm for C153, and *λ*_ex_ = 464 nm for DCM. Data are presented as mean ± standard deviation (*n* = 5).

^b)^ Fluorescence lifetime. *λ*_ex_ = 590 nm for Nile red, *λ*_ex_ = 405 for C153, and *λ*_ex_ = 470 nm for DCM.

^c)^ mono‐exponential fitting.

^d)^ bi‐exponential fitting. Data are presented as mean ± standard deviation (*n* = 3).

Similarly, for C153 and DCM, encapsulation in **Fr‐F6** resulted in increased quantum yields and extended fluorescence lifetimes compared to those in organic solvent. Specifically, the quantum yield of C153 rose from 0.48 in MeOH to 0.73, and its fluorescence lifetime increased from 4.01 to 6.89 ns upon encapsulation in **Fr‐F6** (Table 2; Figure S5c, Supporting Information). DCM also showed an increase in quantum yield from 0.35 in MeOH to 0.41 and an extension in fluorescence lifetime from 1.38 to 2.98 ns in **Fr‐F6** (Table 2; Figure S5d, Supporting Information).

## 3 Discussion

Ferritin cages have been reported to encapsulate various organic molecules and organometallic complexes, including Neutral Red,^[^ ^18^ ^]^ Fluorescein,^[^ ^19^ ^]^ Methylene Blue,^[^ ^20^ ^]^ Hypocrellin,^[^ ^21^ ^]^ and ferrocene derivatives.^[^ ^14a^ ^]^ These encompassed mostly water‐soluble compounds or those modified to enhance water solubility, posing challenges in the encapsulation and precise control of binding sites for hydrophobic organic molecules. To address this, we fabricated a molecular binding pocket at the two‐fold symmetric interface of ferritin by introducing Phe residues at the two‐fold symmetric interface and demonstrated the immobilization of hydrophobic aromatic fluorescent molecules at the binding pocket. This achievement enables the construction of molecular systems driven by molecular fixation and control over the properties of fluorescent molecules within the cage.

**Figure 6:**
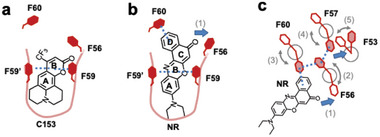
A proposed mechanism of aromatic residue orientation change. a) Ligand binding mode of **C153•Fr‐F6**. b) Ligand binding mode of **NR•Fr‐F6**. Only single conformers of F60 and F56 are shown for clarity. c) Phe side‐chain orientation changes induced by NR binding to **Fr‐F6**. The F59’ represents the F59 from another monomer of the dimer.

Among various combinations of aromatic fluorescent molecules and designed ferritin aromatic variants, cooperative orientation changes of multiple aromatic residues were observed in **NR•Fr‐F6**. The proposed process is as follows (**Figure** **6**b,c): Step 1) The oxazine ring of NR is recognized by F59–F59', leading to its binding in the pocket (Figure 6b). Step 2) To avoid steric hindrance with NR's carbonyl oxygen, F56 prefers alternate conformer indicating reorientation (Figure 6b, arrow 1 and Figure 6c, arrows 1, 2). Step 3) F60 forms CH–π interactions with NR's ring D and the F56 (Figure 6c, arrow 3). Step 4) F57 reorients to interact with F56 (Figure 6c, arrow 4). Step 5) F53 changes orientation to avoid steric hindrance with F57 (Figure 6c, arrow 5). Increase in the occupancies of the alternate conformer of F56 and F60 from apo‐F6 indicate possible reorientation of residues due to interaction with NR. Notably, C153, despite binding to the pocket, does not induce Phe orientation changes like NR, likely due to the absence of aromatic rings C and D in its structure, and its aromatic rings A and B being sufficiently distant from F56 and F60 (Figure 6a,b). While the steps are described sequentially for clarity, the actual time course in this system remains unknown. Given reports of aromatic residue side‐chain orientation changes occurring on the nanosecond scale in NMR and MD simulations of Tyr peptides,^[^ ^22^ ^]^ steps 1 to 5 in this system are anticipated to proceed within a nanosecond scale or longer.

Our primary objective is to control the dynamics of protein sidechains through aromatic interactions triggered by ligand‐binding within the designed pocket. While this is the central focus of this study, we also observed intriguing fluorescent properties of the aromatic ligands attributed to aromatic stacking interactions in the pockets. Nile Red (NR) encapsulated within the F59–F59' pockets of ferritin variants (**Fr‐F1**, **Fr‐F2**, **Fr‐F4**, **Fr‐F6**) demonstrated quantum yields (88–95%) and fluorescence lifetimes (5.2–5.7 ns) surpassing those observed in organic solvents or within supramolecular complexes. This performance notably exceeds the quantum yields reported for NR encapsulated within aromatic micelles (32%)^[^ ^17^ ^]^ and immobilized on laponite nanoclay surfaces (34%),^[^ ^23^ ^]^ and even outperforms NR's fluorescence in favorable organic solvents like dichloromethane, carbon tetrachloride, dioxane, acetone, and acetonitrile, where quantum yields peak at 70–78% with lifetimes of 4.1–4.7 ns.^[^ ^24^ ^]^ Nile Red (NR) is known to readily form aggregates, significantly diminishing its absorbance characteristics in aqueous solutions.^[^ ^25^ ^]^ Remarkably, the ferritin cage encapsulates NR in this system, maintaining quantum yields ≈90% even in an aqueous environment. The enhancement of fluorescence properties within ferritin is attributed to 1) the isolation of fluorescent molecules within the hydrophobic and aromatic environment of the F59–F59' pockets, preventing the formation of quenching H‐aggregates,^[^ ^26^ ^]^ and 2) the planar π–π stacking interactions between F59–F59', which restrain the rotation of NR's dimethylamine groups, thus enhancing the planarity of the NR structure.

A comparison of ferritin variants highlights the crucial role of sandwich‐type π–π stacking interactions between F59–NR–F59' in enhancing fluorescence properties over the mere number of aromatic residues introduced. Notably, **NR•Fr‐F1** and **NR•Fr‐F2**, containing only one and two Phe residues, respectively, achieve higher quantum yields (94 and 95%, respectively) and longer lifetimes (5.54 and 5.75 ns, respectively) than **NR•Fr‐F6** with six Phe residues per monomer (Table 2). Variations in the local amino acid environment surrounding the fluorescent molecule and the orientation of NR's aromatic planes relative to F59 likely account for minor differences in fluorescence properties among the ferritin variants. The hydrophobicity and polarity of the protein surface surrounding NR are known to affect its fluorescence properties.^[^ ^27^ ^]^ The hydrophobic and polar nature of the protein surface around NR influences its fluorescence properties, with the partially polar microenvironment in **Fr‐F1** and **Fr‐F2**, attributed to Glu residues near the pocket entrance, potentially enhancing fluorescence.

The entry pathway of fluorescent molecules into the ferritin aromatic variant cages may involve passage through the two‐fold symmetric interfaces and the canonical three‐fold channels. While ferritin typically utilizes its three‐fold channels, lined with hydrophilic residues such as Glu and Asp, for metal uptake, it is unclear if hydrophobic aromatic molecules like NR, C153, and DCM could traverse these channels. Recent suggestions propose that drug molecules with aromatic structures, like doxorubicin, might access cages via two‐fold symmetric interfaces.^[^ ^28^ ^]^ This implication aligns with our findings, where only variants with engineered pockets at the two‐fold symmetric interfaces successfully encapsulate fluorescent molecules. This comparative analysis, along with prior reports,^[^ ^28^ ^]^ indirectly supports the potential entry of fluorescent molecules through two‐fold symmetric interfaces. Such a possibility invites further exploration through both computational and experimental methods.

## 4 Conclusion

We successfully constructed aromatic clusters with pockets on the internal surfaces of protein cages by introducing phenylalanine (Phe) residues at the two‐fold symmetric interfaces and promoted the encapsulation of hydrophobic aromatic fluorescent molecules (NR, C153, and DCM). In **NR•Fr‐F6**, a conformational change in Phe residues directly interacting with the ligand was observed. Such conformational changes of Phe residues and interaction with ligand indicate the involvement of cooperative effect. This study illustrates the feasibility of controlling protein side chain dynamics remote from the ligand‐binding site, opening avenues for leveraging this phenomenon as a propagation mechanism for molecular behavior, information transfer across nanoscale distances, and spatiotemporal regulation of biological functions. In nature, relay movements of electrons and holes mediated by multiple tyrosine (Tyr) and tryptophan (Trp) residues have been reported in plant photoreceptors.^[^ ^29^ ^]^ Future endeavors to align additional aromatic residues along helices could facilitate long‐distance propagation of molecular behavior, and substituting the constructed Phe clusters with Tyr or Trp residues will lead to novel molecular systems capable of modulating electron transfer properties in response to ligand binding. Our approach broadens the range of molecules that can be encapsulated, including previously inaccessible water‐insoluble aromatic compounds. While biologically relevant molecules such as doxorubicin, which contains aromatic rings, have been encapsulated in ferritin for targeted delivery,^[^ ^30^ ^]^ the engineered protein cages with aromatic pockets developed in this study offer the potential to expand drug delivery applications to encompass a wider variety of hydrophobic aromatic compounds.

## 5 Experimental Section

### Materials and Preparation of Proteins

All reagents were purchased from TCI, Wako, Nacalai Tesque, and Sigma–Aldrich and used as received. Ferritin mutant was prepared using KOD‐Plus‐Mutagenesis kit (Toyobo). The NovaBlue competent cells (Novagen) were transformed with the expression vector pMK2. The *E. coli*. was cultivated and purified by anion‐exchange and size‐exclusion chromatography as previously reported.^[^ ^15^ ^]^ The molecular mass of ferritin mutants was determined by MALDI‐TOF‐MS (Bruker, ultrafleXtreme). The hydrodynamic diameter of ferritin was measured by DLS using Zetasizer Nano (Malvern) at 25 °C with 1 µM of protein solutions prepared in 50 mM Tris‐HCl, pH 8, 150 mM NaCl buffer.

### Crystallization and X‐ray Data Collection

The crystallization of ferritin was performed by the hanging‐drop vapor diffusion method. For apo‐ferritin, the drops were prepared by mixing an equal volume (1.5 µL) of protein solution (15–20 mg mL^−1^, 50 mM Tris‐HCl, pH 8, 150 mM NaCl) and precipitant solution (0.5–1 m (NH_4_)_2_SO_4_, 10–20 mm CdSO_4_ aqueous solution), and equilibrated against the precipitant solution at 20 °C. The crystals were observed within a week.

For ferritin‐fluorescent molecule complex, fluorescent molecules were added to the apo‐ferritin in solution first, followed by crystallization. Specifically, fluorescent molecules (Nile red, Coumarin 153, or DCM, 100 equivalent against ferritin cage) in 100 µL of methanol were mixed with 20 µm ferritin in 100 µL of 50 mM Tris‐HCl, pH 8, 0.15 m NaCl, and incubated at 50 °C for 24 hours. The mixture was dialyzed against 50 mm Tris‐HCl, pH 8, 0.15 M NaCl solution, centrifuged in a benchtop centrifuge at 10 000 rpm for 1 min, and the supernatant was filtrated using a 0.45 µm filter. The resulting ferritin‐fluorescent molecule complex solution was concentrated for the hanging‐drop crystallization. An equal volume (1.5 µL) of protein‐fluorescent molecules solution (15–20 mg mL^−1^, 50 mm Tris‐HCl, pH 8, 150 m NaCl) was mixed with precipitant solution (0.5–1 m (NH_4_)_2_SO_4_, 10–20 m CdSO_4_ aqueous solution), and equilibrated against the precipitant solution at 20 °C.

The X‐ray diffraction data of the ferritin crystals were collected using the single crystal X‐ray diffractometer (XtaLaB Synergy‐DW, Rigaku) at Suzukakedai Materials Analysis Division, Tokyo Institute of Technology. Before data collection, crystals were soaked into the cryoprotectant solution (0.5  Na_2_SO_4_, 20 m CdSO_4_, and 10 m Tris‐HCl) containing 25% (w/w) ethylene glycol and subsequently frozen in liquid nitrogen. The X‐ray diffraction data were collected at –180 °C using an X‐ray wavelength of 1.54 Å. The data were processed using CrysAlisPro in the cubic *F*432 space group. Selected crystallographic parameters are summarized in Tables S1–S3 (Supporting Information).

### X‐ray Data Refinement

The crystal structures of ferritin were determined by molecular replacement method (MOLREP) using the apo‐ferritin structure (PDB ID: 1DAT) as an initial model. The structures were refined by using REFMAC5 in CCP4 suit and rebuilt in COOT using sigma‐weighted (2*F*_o_–*F*_c_) and (*F*_o_–*F*_c_) electron‐density maps. Water molecules were positioned to fit residual (*F*_o_–*F*_c_) density peaks with a lower cutoff of 3σ. The Cd binding sites were assigned by considering the anomalous maps and comparison with reported structures.^[^ ^31^ ^]^ The occupancy values of Cd atoms were adjusted manually by considering the difference map and refined with fractional occupancies. The position of ligands, NR and C153 were verified by omit maps as well as difference density features in comparison with apo‐protein. The models were validated with procheck and wwPDB validation server. The atomic coordinates of the crystal structures were deposited in the Protein Data Bank. The accession codes are given in Tables S1–S3 (Supporting Information).

### Differential Scanning Calorimetry (DSC)

DSC measurements were performed on the MicroCal PEAQ‐DSC (Malvern). The 1 mg mL^−1^ of protein samples were prepared in 50 m HEPES, pH 8, 150  NaCl buffer. The solutions were heated at a scan rate of 1 °C min^−1^ over a temperature range of 50–130 °C under pressure of 4 bar to avoid degassing. Buffer scans were run prior to the sample measurement. The DSC raw data was analyzed using MicroCal software provided by Malvern. After subtracting a buffer–buffer background scan and adjusting the baseline, a plot of heat capacity (*C*_p_) versus temperature was obtained. The experimental curves were deconvoluted to several peaks and were fit with a non‐two‐state model. The median temperatures (*T*_m_) were obtained from the each deconvoluted peaks. The calorimetric enthalpy (Δ*H*) values were calculated from the areas under the unfolding curve.

### Incorporation of Fluorescent Molecules into Protein Cages

Fluorescent molecules (Nile red, Coumarin 153, or DCM, 100 equivalent against ferritin cage) in 100 µL of methanol were mixed with 20 µ ferritin in 100 µL of 50 mM Tris‐HCl, pH 8, 0.15 M NaCl, and incubated at 50 °C for 24 hours. The mixture was dialyzed against 50 m Tris‐HCl, pH 8, 0.15 m NaCl solution, centrifuged in a bench top centrifuge at 10 000 rpm for 1 min and the supernatant was filtrated using 0.45 mm filter. For spectroscopic analysis, the ferritin in complex with fluorescent probes was further purified using size exclusion column (Sephacryl S‐300 HR) equilibrated with 50 m Tris‐HCl, pH 8, 0.15  NaCl buffer.

The encapsulation of fluorescent molecules was analyzed through UV‐vis absorption spectroscopy and high‐performance liquid chromatography (HPLC). For HPLC analysis, a gel filtration column (Asahipack GF‐510HQ, Shodex) was employed, monitoring the UV‐absorbance (280 nm) and fluorescence. The excitation and emission wavelengths for the fluorescence were set at *λ*_ex_ = 535 nm, *λ*_em_ = 600 nm for Nile red; *λ*_ex_ = 394 nm, *λ*_em_ = 480 nm for coumarin 153; and *λ*_ex_ = 464 nm, *λ*_em_ = 560 nm for DCM, respectively.

The number of fluorescent molecules within the protein cage was determined by measuring the UV‐vis absorption spectra of ferritin–fluorescent molecule complex solutions, using molar extinction coefficients of 39600 M^−1^ cm^−1^ at 552 nm for Nile red; 20200 M^−1^ cm^−1^ at 423 nm for C153; and 49700 M^−1^ cm^−1^ at 467 nm for DCM. The protein concentration was determined by Lowry assay, with a detection wavelength of 750 nm.

### Fluorescence Quantum Yield Measurement

The fluorescence quantum yields of the ferritin–fluorophore complexes were measured using an Absolute PL quantum yield system (Hamamatsu C9920‐02G). The excitation wavelengths were set to *λ*_ex_ = 535 nm for Nile red, *λ*_ex_ = 394 nm for coumarin 153, and *λ*_ex_ = 464 nm for DCM, respectively.

### Fluorescence Lifetime Measurement

The fluorescence lifetime of the ferritin–fluorophore complexes were measured using Hamamatsu C7700‐ABS‐N. The excitation and emission wavelengths were set to *λ*_ex_ = 590 nm, *λ*_em_ = 630 nm for NR•ferritin complex; *λ*_ex_ = 590 nm, *λ*_em_ = 630 nm for NR in MeOH. *λ*_ex_ = 405 nm, *λ*_em_ = 480 nm for CMR•ferritin complex; *λ*_ex_ = 405 nm, *λ*_em_ = 550 nm for CMR in MeOH. *λ*_ex_ = 470 nm, *λ*_em_ = 560 nm for DCM•ferritin complex; *λ*_ex_ = 470 nm, *λ*_em_ = 640 nm for DCM in MeOH, respectively.

### Statistical Analysis

Quantification of the number of fluorescent molecules incorporated into a ferritin cage (Table 1), fluorescence quantum yield measurement (Table 2), fluorescence lifetime measurement (Table 2), DLS measurement (Figure S1b, Supporting Information), and DSC (Figure S2h, Supporting Information) were conducted in replicates, with sample size (*n*) indicated in the corresponding figure and table legends. Data are presented as mean ± standard deviation (SD).

## Conflict of Interest

The authors declare no conflict of interest.

## Author Contributions

Y.H., T.S., and B.M. contributed equally to this work. Y.H. and T.U. wrote the manuscript. H.N. and A.A. prepared the plasmids of ferritin mutants. Y.H., H.N., and A.A. performed protein production and characterization. Y.H. solved the crystal structures. Y.H. and T.S. refined the crystal structures with assistance from B.M. and S.A. Y.H. conducted the thermal measurements with assistance from S.N. and K.T. in differential scanning calorimetry. Y.H. performed incorporation of fluorophores and spectroscopic characterization with assistance from M.Y. and Y.K. in fluorescence measurements. T.U. supervised the project.

## Supporting information



Supporting Information

## Data Availability

The data that support the findings of this study are available in the supplementary material of this article.
