# Supplementary material for: Design of Aromatic Interaction Networks in a Protein Cage Modulated by Fluorescent Ligand Binding
Source: Adv Sci (Weinh). 2025 Feb 20;12(15):2417030. doi: 10.1002/advs.202417030 (PMC12005729; doi:10.1002/advs.202417030)
Supplement: Supplementary file 1 — Supporting Information [file ADVS-12-2417030-s001.pdf]

ADVANCED SCIENCE
Open Access

Supporting Information

for *Adv. Sci.*, DOI 10.1002/advs.202417030

Design of Aromatic Interaction Networks in a Protein Cage Modulated by Fluorescent Ligand Binding

*Yuki Hishikawa, Taiga Suzuki, Basudev Maity, Hiroki Noya, Michito Yoshizawa, Asuka Asanuma, Yuri Katagiri, Satoshi Abe, Satoru Nagatoishi, Kouhei Tsumoto and Takafumi Ueno**

Supplementary Information

Design of Aromatic Interaction Networks in a Protein Cage Modulated by Fluorescent Ligand Binding

Yuki Hishikawa,[a,f] Taiga Suzuki,[a,f] Basudev Maity,[a,f] Hiroki Noya,[a] Michito Yoshizawa,[b] Asuka Asanuma,[a] Yuri Katagiri,[b] Satoshi Abe,[a] Satoru Nagatoishi,[c] Kouhei Tsumoto,[c,d] Takafumi Ueno*[a,e]

[a] Dr. Y. Hishikawa, H. Noya, T. Suzuki, Dr. B. Maity, A. Asanuma, Dr. S. Abe, Prof. T. Ueno
School of Life Science and Technology, Institute of Science Tokyo
Nagatsuta-cho 4259, Midori-ku, Yokohama 226-8501, Japan
E-mail: tueno@bio.titech.ac.jp

[b] Prof. M. Yoshizawa, Yuri Katagiri
Laboratory for Chemistry and Life Science, Institute of Integrated Research, Institute of Science Tokyo
Nagatsuta-cho 4259, Midori-ku, Yokohama 226-8501, Japan

[c] Assoc. Prof. S. Nagatoishi, Prof. K. Tsumoto
The Institute of Medical Science, The University of Tokyo
Shirokanedai 4-6-1, Minato-ku, Tokyo 108-8639, Japan

[d] Prof. K. Tsumoto
Department of Bioengineering, School of Engineering, The University of Tokyo
Hongo 7-3-1, Bunkyo-ku, Tokyo 113-8656, Japan

[e] Prof. T. Ueno
Research Center for Autonomous Systems Materialogy (ASMat), Institute of Integrated Research, Institute of Science Tokyo
Nagatsuta-cho 4259, Midori-ku, Yokohama 226-8501, Japan

[f] Equal contribution status

* Correspondence to: tueno@bio.titech.ac.jp

1. Supplementary figures

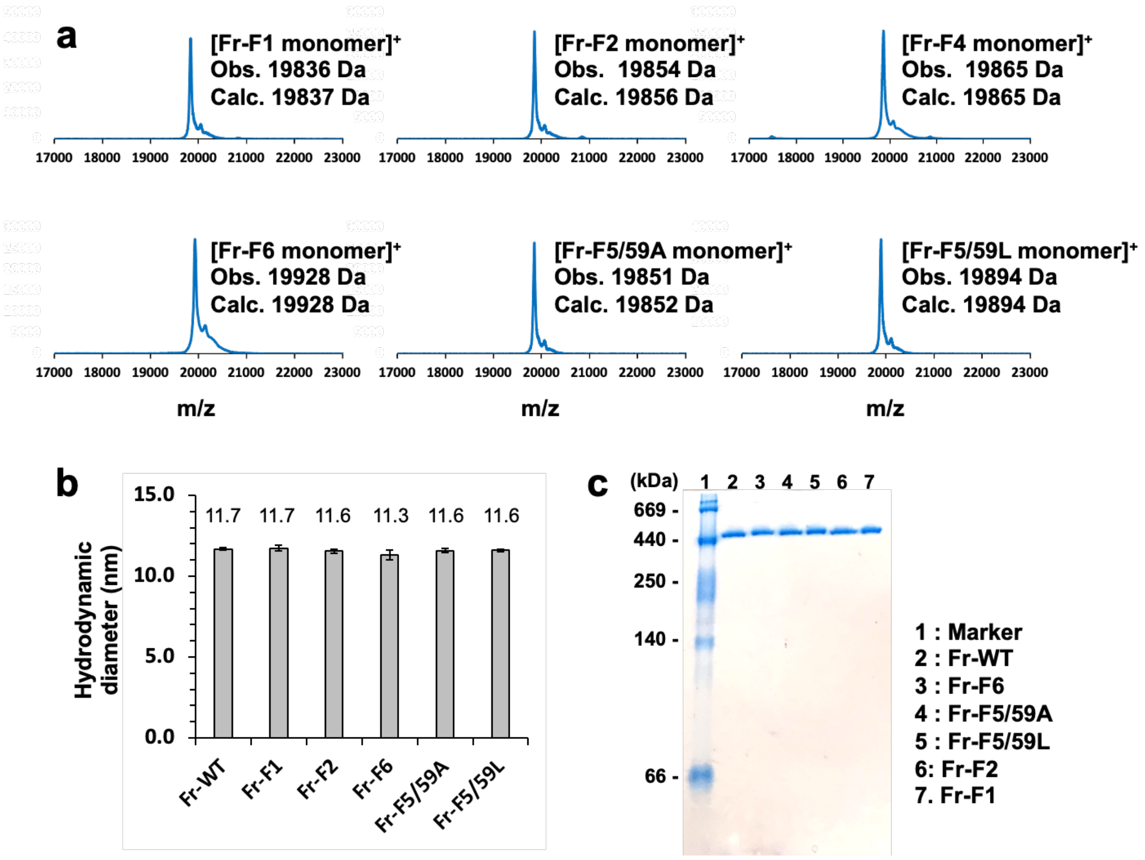

Figure S1. Physicochemical characterization of ferritin. (a) MALDI-TOF-MS spectra. The observed mass was 19836 Da (calc. 19837 Da) for **Fr-F1**, 19854 (calc. 19856 Da) for **Fr-F2**, 19865 Da (calc. 19865 Da) for **Fr-F4**, 19928 Da (calc. 19928 Da) for **Fr-F6**, 19851 Da (calc. 19852 Da) for **Fr-F5/59A**, and 19894 Da (calc. 19894 Da) for **Fr-F5/59L**. (b) Dynamic light scattering (DLS) data showing the hydrodynamic diameter of the ferritin mutants in 50mM Tris-HCl buffer (pH8.0) containing 150 mM NaCl. The error bars represent standard deviation (n = 6). (c) Native PAGE banding pattern of FrWT and mutants showing the formation of 24-mer cage.

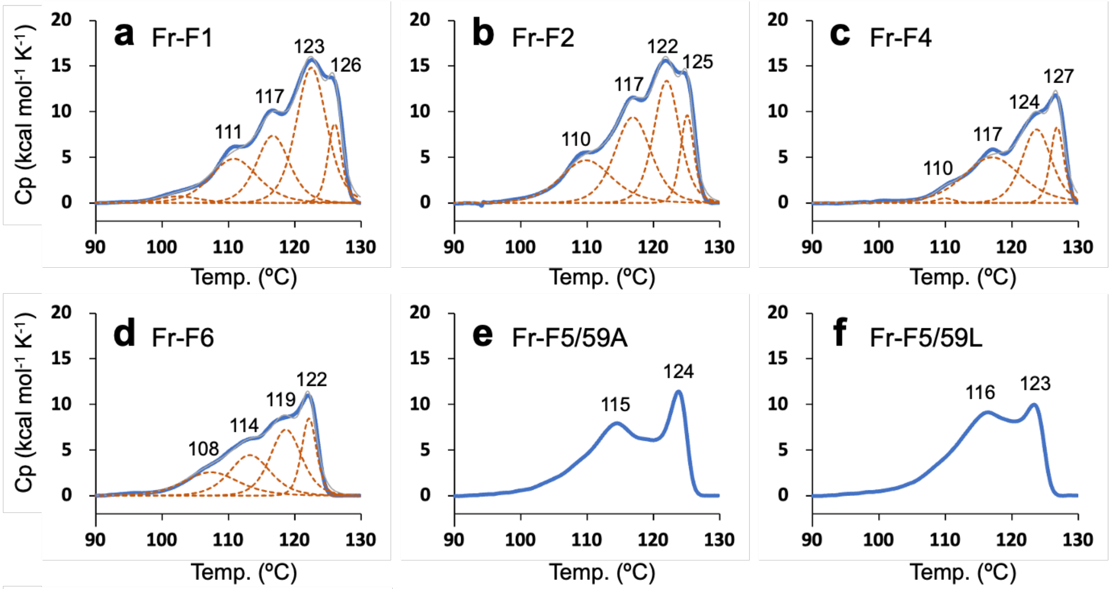

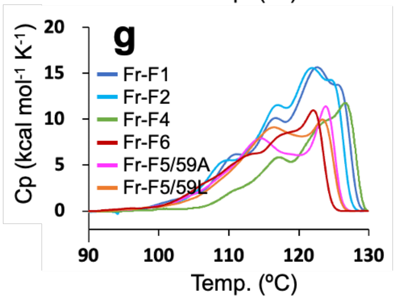

h

	T_m (ºC)	ΔH (kcal mol^{-1})
Fr-F1	125.9 ± 0.1	205 ± 13
Fr-F2	125.1 ± 0.1	213 ± 4
Fr-F4	126.9 ± 0.1	120 ± 14
Fr-F6	122.2 ± 0.1	130 ± 11
Fr-F5/59A	123.8 ± 0.0	139 ± 4
Fr-F5/59L	123.2 ± 0.2	155 ± 19

Figure S2. Evaluation of thermostability of ferritin by DSC. (a, b, c) DSC thermogram with deconvoluted peaks. **Fr-F1** (a), **Fr-F2** (b), **Fr-F4** (c), **Fr-F6** (d), **Fr-F5/59A** (e), **Fr-F5/59L** (f). In panels a-f, the solid blue lines represent the experimentally measured curves, while the dashed orange lines denote the deconvoluted peaks. The numbers associated with each peak correspond to the melting temperatures (T_m) in degrees Celsius (ºC). The thermograms for panel e (Fr-F5/59A) and panel f (Fr-F5/59L) exhibit shapes suggesting multiple peaks buried between the two primary peaks, which could not be separated through deconvolution analysis. (g) the overlay of the differential scanning calorimetry (DSC) thermograms for each variant. (h) denaturation temperature (T_m) and the change in denaturation enthalpy (ΔH) for the ferritin variants, indicating the mean values and standard deviations. The sample sizes are n = 3 for **Fr-F1**, **Fr-F4**, and **Fr-F6**, and n = 2 for **Fr-F2**, **Fr-F5/59A**, and **Fr-F5/59L**. The DSC measurements were conducted with a protein concentration of 1 mg mL^{-1}, in 50 mM HEPES (pH 8), 150 mM NaCl, a temperature range of 50–130 ºC, and a heating rate of 1 ºC min^{-1}.

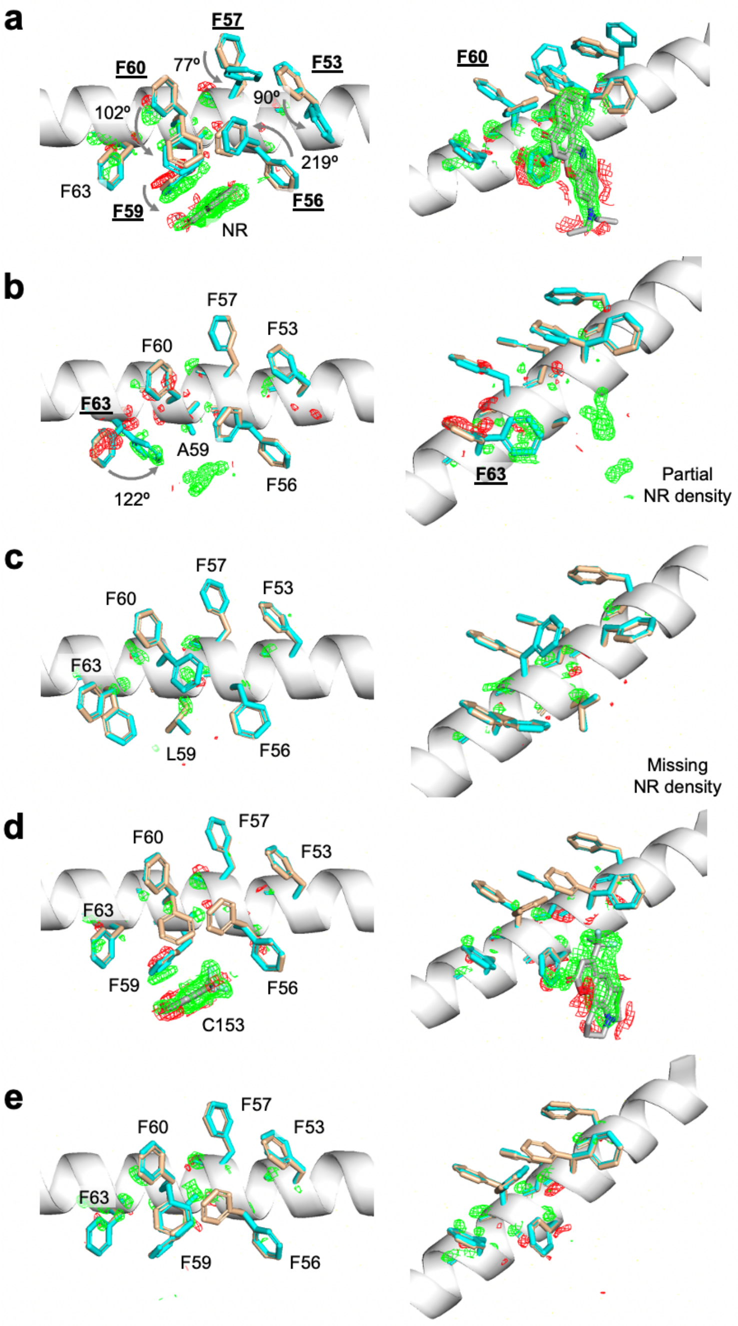

Figure S3. Comparison of aromatic side chain orientations in **Fr-F6**, **Fr-F5/59A**, and **Fr-F5/59L** before and after substrate binding. (a) Structures of **Fr-F6** and **NR•Fr-F6**. (b) Structures of **Fr-F5/59A** and **NR•Fr-F5/59A**. (c) Structures of **Fr-F5/59L** and **NR•Fr-F5/59L**. (d) Structures of **Fr-F6** and **C153•Fr-F6**. (e) Structures of **Fr-F6** and **DCM•Fr-F6**. In panels a–e, side chain orientations before the binding are shown in orange, and those after the binding in cyan. Difference Fourier maps F_o(NR•Fr-Fx) - F_o(Fr-Fx) (3 σ) show increased positive electron density in green and decreased negative electron density in red mesh. Residues with significant orientation changes upon the NR binding are highlighted with underlines.

a b c d

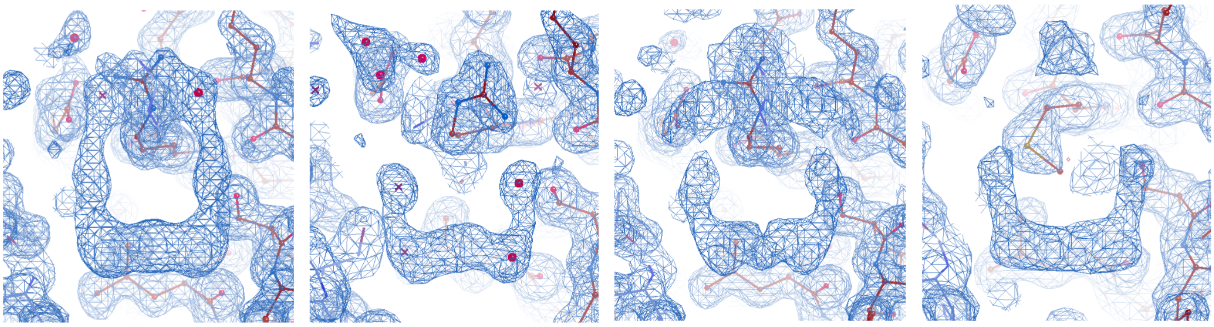

Figure S4. Typical electron density ($2F_o$-F_c at 1σ contour) maps at the 2-fold symmetric interface of the selected ferritin cages from reported PDB structures with accession code (a) 2W0O, (b) 3F32, (c) 2V2M, and (d) 2V2O. No ligand was reacted with ferritin in these structures.

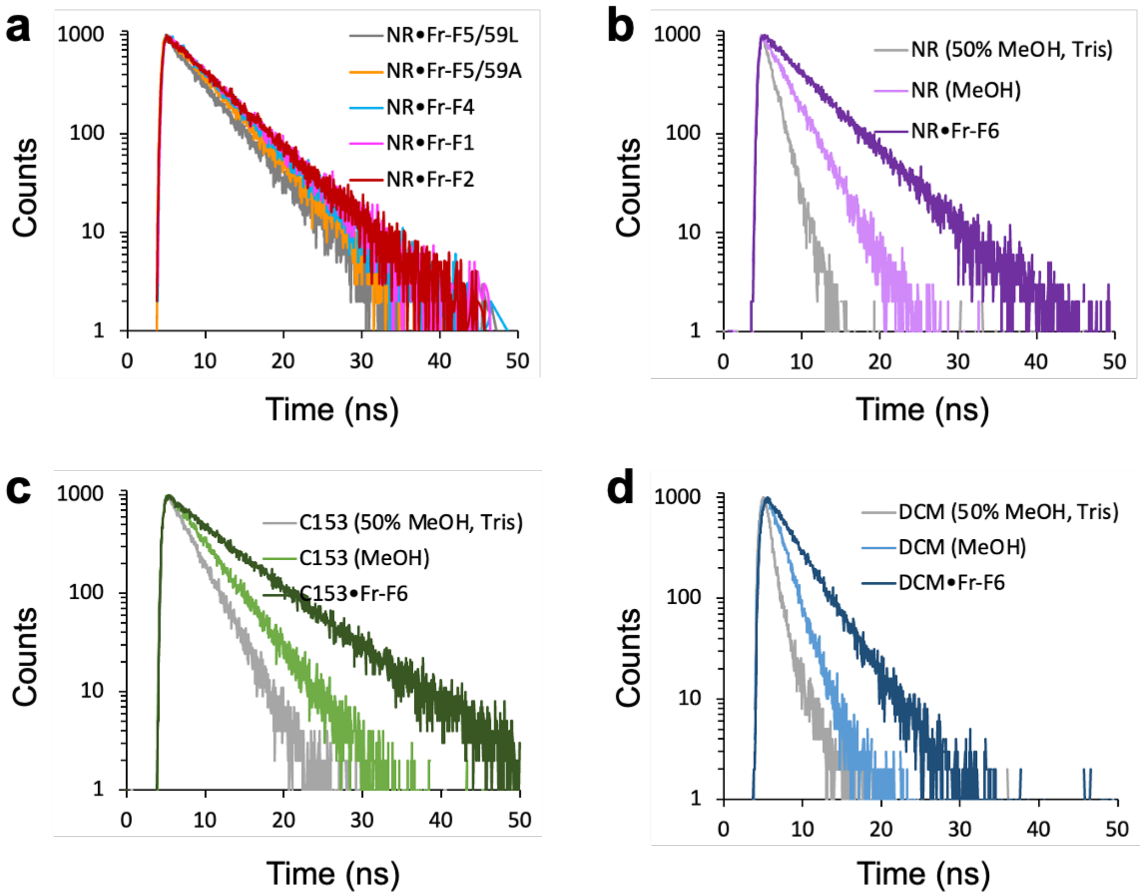

Figure S5. Fluorescence decay curves of fluorescent molecules in complex with ferritin or in methanol. (a) NR in **Fr-F1**, **Fr-F2**, **Fr-F4**, **Fr-F5/59A**, and **Fr-F5/59L**. (b) NR in **Fr-F6**, methanol, and methanol-Tris mixed solvents. (c) C153 in **Fr-F6**, methanol, and methanol-Tris mixed solvents. (d) DCM in **Fr-F6**, methanol, and 50% methanol-Tris mixed solvents. λ_{ex} = 590 nm for Nile red, λ_{ex} = 405 for C153, and λ_{ex} = 470 nm for DCM.

2. Supplementary tables

Table S1. Summary of X-ray data collection and refinement statistics.

	**Fr-F1**	**Fr-F2**	**Fr-F6**	**Fr-F5/59A**	**Fr-F5/59L**
PDB ID	9KPA	9KP5	9KKR	9KRS	9KN7
**Data collection**					
X-ray wavelength (Å)	1.54	1.54	1.54	1.54	1.54
Space group	*F*432	*F*432	*F*432	*F*432	*F*432
Cell dimensions					
$a = b = c$ (Å)	180.84	180.93	181.43	181.19	180.72
$\alpha = \beta = \gamma$ (°)	90	90	90	90	90
Resolution range (Å) [a]	18.46–1.50 (1.53–1.50)	18.97–1.50 (1.53–1.50)	19.91–1.50 (1.53–1.50)	18.49–1.53 (1.56–1.53)	18.94–1.50 (1.53–1.50)
Completeness (%) [a]	99.9 (99.8)	100.0 (100.0)	99.9 (99.2)	99.9 (100.0)	99.9 (100.0)
Multiplicity [a]	10.1 (6.7)	10.2 (6.8)	12.4 (8.0)	12.8 (8.7)	9.9 (6.7)
Unique reflections [a]	40986 (2014)	41041 (1999)	41371 (1999)	38887 (1880)	40895 (1981)
R_{merge} [a]	0.046 (0.351)	0.044 (0.331)	0.063 (0.717)	0.054 (0.832)	0.042 (0.362)
R_{meas} [a]	0.051 (0.414)	0.049 (0.390)	0.068 (0.820)	0.059 (0.937)	0.046 (0.429)
I/σ (I) [a]	30.8 (4.7)	33.2 (4.9)	24.5 (2.3)	30.8 (2.4)	31.8 (4.5)
CC(1/2) [a]	1.000 (0.938)	1.000 (0.948)	0.999 (0.867)	1.000 (0.813)	1.000 (0.941)
**Refinement**					
Resolution (Å)	1.50	1.50	1.50	1.53	1.50
Reflections used	40900	40995	40919	38816	40810
R_{work} / R_{free} [b]	0.180 / 0.194	0.178 / 0.188	0.185 / 0.203	0.184 / 0.212	0.165 / 0.178
r. m. s. deviations					
Bond lengths (Å)	0.016	0.016	0.013	0.016	0.013
Bond angles (°)	1.737	1.689	1.649	1.719	1.741
Ramachandran plot [c]					
Favored	167	167	169	169	169
Allowed	3	4	3	3	3
Outlier	0	0	0	0	0

[a] Values in parentheses are for the highest resolution shell.

[b] $R_{work} = \sum||F_o| - |F_c||/\sum|F_o|$, where F_o and F_c are the observed and calculated structure factor amplitudes, respectively. R_{free} is an R factor calculated on a partial set that is not used in the refinement of the structure.

[c] Ramachandran plot parameters were calculated using *RAMPAGE*.

Table S2. Summary of X-ray data collection and refinement statistics

	**NR• Fr-F1**	**NR• Fr-F2**	**NR• Fr-F4**	**NR• Fr-F6**
PDB ID	9KP7	9KP2	9KLE	9KKP
**Data collection**				
X-ray wavelength (Å)	1.54	1.54	1.54	1.54
Space group	*F*432	*F*432	*F*432	*F*432
Cell dimensions				
$a = b = c$ (Å)	181.33	180.91	181.55	181.51
$\alpha = \beta = \gamma$ (°)	90	90	90	90
Resolution range (Å) [a]	19.01–1.50 (1.53–1.50)	18.97–1.50 (1.53–1.50)	19.03–1.65 (1.68–1.65)	17.80–1.60 (1.63–1.60)
Completeness (%)[a]	100.0 (100.0)	99.9 (100.0)	99.9 (100.0)	99.9 (100.0)
Multiplicity [a]	9.9 (6.4)	10.2 (6.8)	10.1 (6.6)	10.0 (6.3)
Unique reflections [a]	41305 (2015)	41026 (1995)	31335 (1486)	34271 (1662)
R_{merge} [a]	0.060 (0.587)	0.049 (0.459)	0.071 (0.773)	0.049 (0.466)
R_{meas} [a]	0.066 (0.696)	0.054 (0.542)	0.079 (0.918)	0.054 (0.553)
I/σ (I) [a]	23.1 (2.7)	28.2 (3.6)	22.1 (2.1)	29.8 (3.4)
CC(1/2) [a]	0.999 (0.856)	1.000 (0.911)	0.999 (0.823)	1.000 (0.904)
**Refinement**				
Resolution (Å)	1.50	1.50	1.65	1.60
Reflections used	41197	40952	30993	32413
R_{work} / R_{free} [b]	0.178 / 0.196	0.178 / 0.190	0.191 / 0.209	0.164 / 0.186
r. m. s. deviations				
Bond lengths (Å)	0.016	0.016	0.013	0.013
Bond angles (°)	1.738	1.651	1.784	1.953
Ramachandran plot [c]				
Favored	168	167	167	168
Allowed	3	3	4	3
Outlier	0	0	0	0

[a] Values in parentheses are for the highest resolution shell.

[b] $R_{work} = \sum||F_o| - |F_c||/\sum|F_o|$, where F_o and F_c are the observed and calculated structure factor amplitudes, respectively. R_{free} is an R factor calculated on a partial set that is not used in the refinement of the structure.

[c] Ramachandran plot parameters were calculated using *RAMPAGE*.

Table S3. Summary of X-ray data collection and refinement statistics

	**NR• Fr-F5/59A**	**NR• Fr-F5/59L**	**C153• Fr-F6**	**DCM• Fr-F6**
PDB ID	**-**	**-**	**9KN8**	**-**
**Data collection**				
X-ray wavelength (Å)	1.54	1.54	1.54	1.54
Space group	*F*432	*F*432	*F*432	*F*432
Cell dimensions				
$a = b = c$ (Å)	181.24	181.69	181.16	181.79
$\alpha = \beta = \gamma$ (°)	90	90	90	90
Resolution range (Å) [a]	19.00–1.60 (1.63–1.60)	19.05–1.66 (1.69–1.66)	18.21–1.64 (1.67–1.64)	18.55–1.62 (1.65–1.62)
Completeness (%)[a]	99.9 (99.4)	99.7 (97.4)	99.5 (99.4)	99.9 (100.0)
Multiplicity [a]	9.8 (6.3)	8.7 (5.1)	8.4 (5.2)	10.4 (6.6)
Unique reflections [a]	34112 (1647)	30789 (1447)	31536 (1587)	33205 (1607)
R_{merge} [a]	0.044 (0.447)	0.047 (0.614)	0.063 (0.791)	0.062 (0.700)
R_{meas} [a]	0.048 (0.534)	0.053 (0.742)	0.071 (0.960)	0.068 (0.824)
I/σ (I) [a]	32.2 (3.4)	27.4 (2.4)	20.9 (2.1)	26.5 (2.4)
CC(1/2) [a]	1.000 (0.894)	0.999 (0.867)	0.999 (0.819)	0.999 (0.801)
**Refinement**				
Resolution (Å)	1.60	1.66	1.64	1.62
Reflections used	34048	30623	31414	33180
R_{work} / R_{free} [b]	0.176 / 0.192	0.184 / 0.177	0.193 / 0.208	0.178 / 0.174
r. m. s. deviations				
Bond lengths (Å)	0.016	0.017	0.015	0.016
Bond angles (°)	1.821	1.868	1.729	1.803
Ramachandran plot [c]				
Favored	168	167	168	168
Allowed	4	4	3	4
Outlier	0	0	0	0

[a] Values in parentheses are for the highest resolution shell.

[b] $R_{work} = \sum||F_o| - |F_c||/\sum|F_o|$, where F_o and F_c are the observed and calculated structure factor amplitudes, respectively. R_{free} is an R factor calculated on a partial set that is not used in the refinement of the structure.

[c] Ramachandran plot parameters were calculated using *RAMPAGE*.
